# Post-transplant acute pancreatitis after pancreas transplantation: application of the International Study Group for Pancreatic Surgery definition and clinical impact in a multicenter cohort study

**DOI:** 10.3389/ti.2026.16849

**Published:** 2026-09-08

**Authors:** Stefano Partelli, Valentina Andreasi, Matteo Santangelo, Oriana Ciacio, Rossana Caldara, Arianna Bertuol, Valentina Tomajer, Davide Catarinella, Alessandra Ida, Caterina Di Bella, Gabriella Pittau, Chady Salloum, Gioia Sgrinzato, Paolo Rigotti, Lorenzo Piemonti, Antonio Sa Cunha, Lucrezia Furian, Massimo Falconi

**Affiliations:** 1 Pancreatic and Transplant Surgery Unit, IRCCS San Raffaele Hospital, Milan, Italy; 2 Vita-Salute San Raffaele University, Milan, Italy; 3 Kidney and Pancreas Transplantation Unit, Padua University Hospital, Padua, Italy; 4 Hôpital Universitaire Paul Brousse, Université Paris-Saclay, Centre Hépato-biliaire, Villejuif, France; 5 Clinic Unit of Regenerative Medicine and Organ Transplants, IRCCS San Raffaele Hospital, Milan, Italy

**Keywords:** amylase, graft pancreatitis, graft survival, hyperamylasemia, pancreas transplantation, post-transplant acute pancreatitis, postoperative complications

## Abstract

Graft pancreatitis is a clinically relevant but poorly standardized complication after pancreas transplantation (PT). In contrast, post-pancreatectomy acute pancreatitis has been formally defined and validated by the International Study Group for Pancreatic Surgery (ISGPS). This study assessed the clinical applicability of an adapted ISGPS-based framework to define post-transplant acute pancreatitis (PTAP) after PT and its association with clinically relevant outcomes. Consecutive patients undergoing PT at three European centers between 2010 and 2024 were included. PTAP was defined by sustained postoperative hyperamylasemia (POH) for ≥48 h combined with CT findings consistent with pancreatitis within 30 days after transplantation. Imaging was performed in 89% of patients based on clinical indication and center-specific protocols. Among 432 patients, the adapted definition was applicable in 416. Sustained POH occurred in 196 patients (47%), and PTAP was diagnosed in 86 (21%). PTAP was the only independent predictor of severe postoperative complications (OR, 2.482; P = 0.001), was independently associated with prolonged hospital stay (OR, 3.817; P < 0.001), and with worse death-censored graft survival (P < 0.001). Cold ischemia time was the only independent determinant of PTAP (P < 0.001). PTAP was a frequent and clinically meaningful complication after PT, occurring in approximately one in five patients and associated with worse short- and long-term outcomes.

## Graphical Abstract

**Figure:**
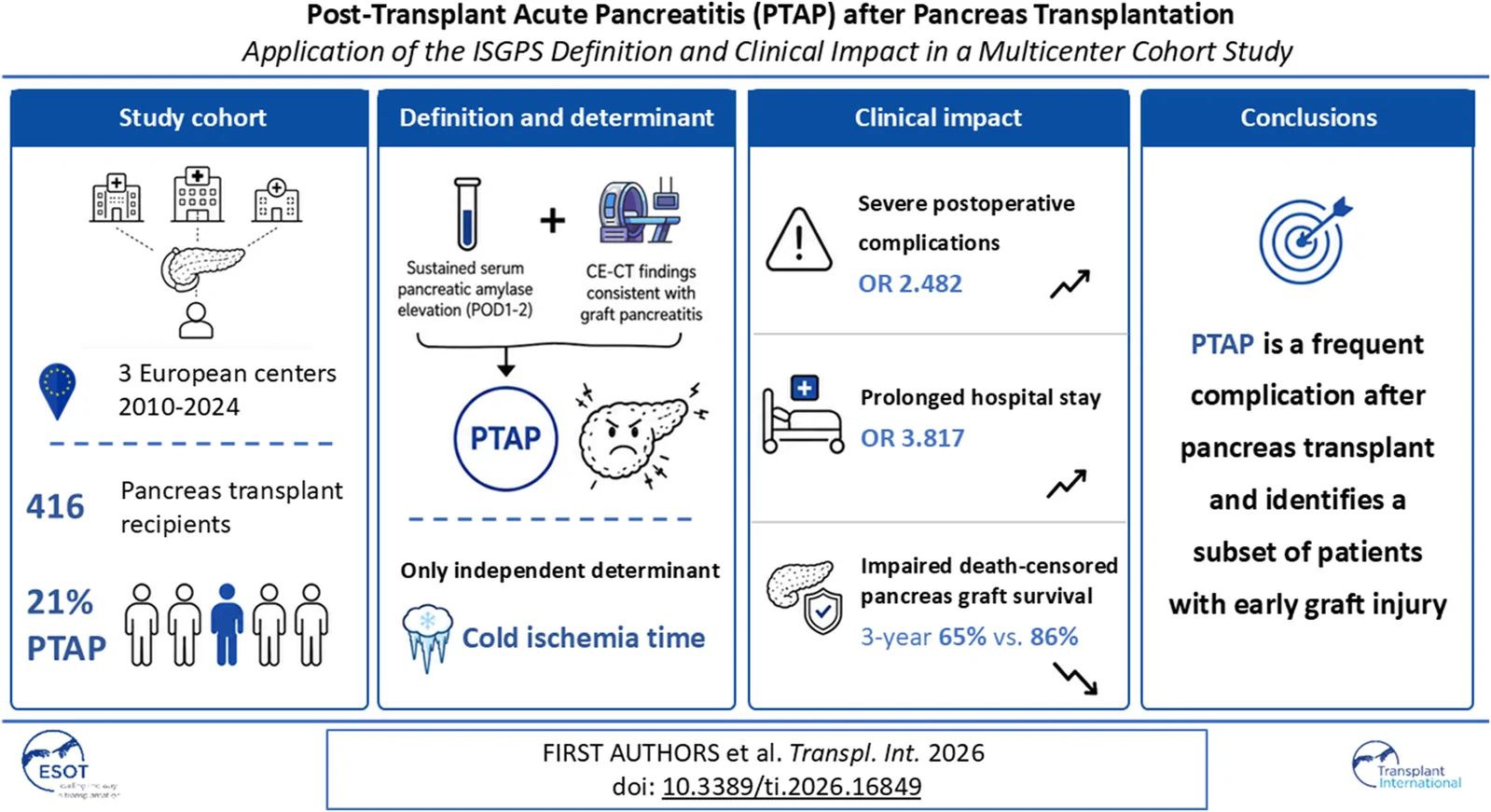

## Introduction

Graft pancreatitis (GP) is a clinically relevant complication after pancreas transplantation (PT), and has been associated with increased postoperative morbidity, including early relaparotomy and graft loss [1, 2]. However, GP remains a poorly standardized clinical entity that encompasses a broad spectrum of pancreatic graft injury. Multiple mechanisms have been implicated in its development, including ischemia-reperfusion injury and technical or preservation-related factors, such as the use of histidine tryptophan ketoglutarate (HTK) solution, graft overflushing, and exocrine bladder drainage [3, 4]. Donor-related factors may also contribute. As a result, both clinical management and outcome reporting remain inconsistent across transplant programs.

To address this gap, a recent scoping review systematically examined the definitions of GP reported in the literature [5]. Twenty studies were identified, each using a different definition based on variable combinations of clinical, biochemical, radiological, and pathological criteria [5]. This lack of standardization is reflected in the wide variation in reported GP rates, ranging from 0% to 87%, which limits meaningful comparisons of postoperative outcomes across institutions [5]. Importantly, the definitions proposed in the transplant setting remain circumstantial and lack formal validation.

The established definition of native acute pancreatitis [6] cannot be directly extrapolated to the surgical and immunological context of PT because of the unique contribution of perioperative ischemia-reperfusion injury, graft denervation, and immunosuppression-related factors. In contrast, the International Study Group for Pancreatic Surgery (ISGPS) has recently proposed and validated a standardized definition of post-pancreatectomy acute pancreatitis (PPAP), based on persistent elevation of serum pancreatic amylase above the institutional upper limit of normal (ULN) for at least 48 h postoperatively and radiological evidence of pancreatitis [7, 8]. Although the mechanisms of pancreatic injury differ substantially between PT and pancreatic resection, both settings are characterized by early perioperative pancreatic injury that may range from transient biochemical abnormalities to clinically relevant inflammatory damage with prognostic implications. Because the ISGPS framework relies on objective biochemical and radiological criteria rather than on a specific etiology [7, 8], it may be applicable to the perioperative setting of PT and could provide a standardized basis for defining post-transplant acute pancreatitis (PTAP).

The aim of this study was to assess the clinical applicability of an adapted ISGPS-based framework in a multicenter cohort of PT recipients to define PTAP and to evaluate its association with clinically relevant short- and long-term outcomes, including postoperative morbidity and pancreas graft survival.

## Materials and methods

### Study design and participants

**FIGURE 1:**
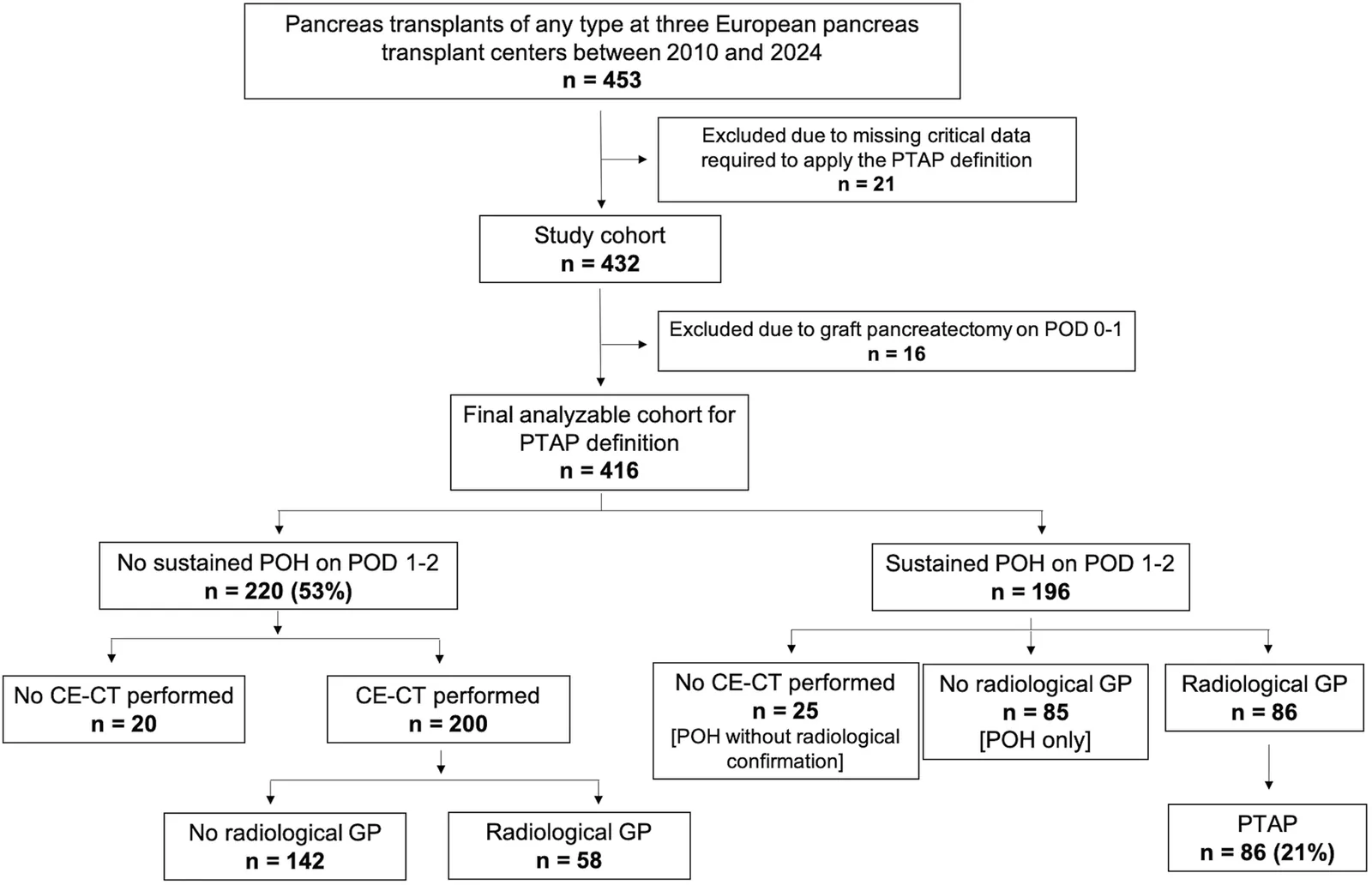
Study flow diagram illustrating patient screening, exclusions, and classification according to sustained postoperative hyperamylasemia and radiological graft pancreatitis. Patients undergoing graft pancreatectomy on postoperative day (POD) 0–1 were excluded because sustained postoperative hyperamylasemia over 48 h could not be assessed, and the adapted ISGPS-based definition of post-transplant acute pancreatitis (PTAP) could therefore not be applied. CE-CT, contrast-enhanced computed tomography; GP, graft pancreatitis; POD, postoperative day; POH, postoperative hyperamylasemia; PTAP, post-transplant acute pancreatitis.

This multicenter retrospective observational study was conducted in accordance with the Strengthening the Reporting of Observational Studies in Epidemiology (STROBE) guidelines [9]. Consecutive patients undergoing PT, including simultaneous pancreas-kidney (SPK), pancreas-after-kidney (PAK), and pancreas transplant alone (PTA), at three European pancreas transplant centers between 2010 and 2024 were considered for inclusion. Patients with missing data required to apply the PTAP definition, including postoperative serum amylase measurements, were excluded. Patients undergoing graft explantation on postoperative day (POD) 0 or 1 were also excluded because sustained postoperative hyperamylasemia could not be assessed and the adapted ISGPS-based definition could therefore not be applied. The patient selection process is summarized in Figure 1.

The study was conducted in accordance with the Declaration of Helsinki. Given the retrospective observational design and the use of anonymized routinely collected clinical data, approval by the local ethics committees was waived at all participating centers.

### PTAP definition

PTAP was defined using an adapted ISGPS framework for PPAP [7, 8]. Specifically, PTAP was defined by the presence of both of the following objective criteria:

1. Sustained postoperative hyperamylasemia (POH): persistent elevation of serum pancreatic amylase above the institutional upper limit of normal (Supplementary Table 1) for at least 48 h postoperatively, defined by elevated values on both POD1 and POD2;
2. Radiological evidence of GP: findings consistent with pancreatitis on contrast-enhanced computed tomography (CE-CT) performed within 30 days after transplantation. Radiological GP was defined by the presence of at least 1 of the following features: interstitial parenchymal edema, inflammatory changes in the peripancreatic graft fat, intra- and/or peripancreatic graft fluid collections, or parenchymal and/or peripancreatic graft necrosis.

Postoperative CE-CT scans were interpreted locally by radiologists at each participating center as part of routine clinical care. No central radiological review was performed. Because imaging was obtained and interpreted in the clinical setting, radiologists were not systematically blinded to serum amylase values or clinical information. For study purposes, PTAP status was assigned retrospectively according to prespecified biochemical and radiological criteria.

Serum pancreatic amylase was routinely measured on POD1 and POD2 in all patients. CE-CT was not performed systematically in all patients with sustained POH but was obtained according to clinical indication or center-specific protocols. CE-CT was the standard postoperative cross-sectional imaging modality across the three participating centers and was performed according to center-specific imaging strategies. In one center, CE-CT was performed according to clinical indication, whereas the other two centers adopted protocol-driven CE-CT for all PT recipients, implemented from 2013 onward in one center and routinely performed throughout the study period in the other. Patients with sustained POH who did not undergo postoperative cross-sectional imaging were classified as having POH without radiological confirmation, because the radiological criterion for PTAP could not be assessed. These patients were therefore considered not to fulfill the PTAP definition, but were not regarded as radiologically negative. This pragmatic imaging strategy, which reflects real-world clinical practice, may have led to underdiagnosis of mild or subclinical forms of PTAP but was expected to enhance specificity for clinically meaningful PTAP, which was the primary focus of the present study.

The clinical criterion included in the original ISGPS definition, namely clinically relevant changes in postoperative management, was intentionally not applied. This choice was made to avoid incorporation bias, because postoperative management decisions are intrinsically linked to short-term clinical outcomes, which constituted primary study endpoints. By relying exclusively on objective biochemical and radiological criteria, the analysis aimed to preserve independence between exposure (PTAP) and outcome assessment.

### Definition of clinical outcomes

The clinical impact of PTAP was evaluated using two primary outcomes: (i) severe postoperative complications and (ii) length of index hospital stay (LOS). Postoperative complications occurring within 30 days after transplantation or during the index hospitalization were graded according to the Clavien-Dindo classification [10] and were considered severe if grade IIIA or higher. This threshold was selected to capture complications of clear clinical relevance requiring invasive management. Both systemic and pancreas-related complications were included, whereas complications exclusively related to the kidney graft were excluded. LOS was calculated from the day of transplantation (POD0) to hospital discharge and dichotomized using the third quartile of the cohort distribution; prolonged LOS was defined as more than 23 days.

Secondary outcomes included death-censored pancreas graft survival (GS) and patient survival. Death-censored pancreas GS was defined as the time from transplantation to pancreas graft loss from any cause, excluding death with a functioning graft. Pancreas graft loss was defined as graft explantation or persistent return to insulin dependence during follow-up. Temporary insulin therapy during the early postoperative period was not considered graft loss if graft endocrine function subsequently recovered. Patient survival was defined as the time from transplantation to death from any cause. Patients without events were censored at last available follow-up.

### Data collection

Clinical data were retrospectively collected from prospectively maintained institutional databases using standardized data collection procedures. Collected variables included recipient- and donor-related intraoperative and postoperative characteristics.

Recipient-related variables included sex, age at transplantation, body mass index (BMI), duration and type of diabetes, comorbidities, preoperative dialysis status and modality, and history of previous transplants. Donor-related variables included donor type (deceased donor after brain death [DBD] or after circulatory death [DCD]), sex, age and BMI.

Intraoperative variables collected included transplant type (SPK, PAK, PTA), cold ischemia time (CIT), anastomotic time, duration of surgery, and intraoperative blood transfusions. CIT was defined as the interval between initiation of cold perfusion in the donor and removal of the graft from ice for implantation. Anastomotic time was defined as the interval between the end of cold preservation and restoration of graft blood flow in the recipient.

Postoperative variables included early and late complications, relaparotomy, hemorrhage, thrombosis (occurring from POD2 onwards), graft explantation, duodenal leakage, and graft rejection before discharge. Clinically relevant graft exocrine leakage was captured as duodenal leakage and recorded as a distinct graft-related complication. Duodenal leakage was defined on the basis of clinical findings, drain characteristics including suspicious drain output and increased drain amylase levels, operative findings, and radiological findings when CT imaging was available. Complications occurring within 30 days after surgery were classified as early, whereas those occurring between 30 days and 3 months were classified as late. Long-term outcomes included rejection episodes, graft loss (with or without graft pancreatectomy), and patient survival.

### Immunosuppression and anticoagulation

Induction immunosuppression consisted of rabbit anti-thymocyte globulin (rATG) and corticosteroids in all patients. Maintenance immunosuppression consisted of a triple-drug regimen including tacrolimus, mycophenolate mofetil (MMF), and corticosteroids.

Perioperative anticoagulation protocols differed across centers and over time, and included prophylactic-dose low molecular weight heparin (LMWH), therapeutic-dose LMWH, or continuous intravenous unfractionated heparin (UFH), with escalation to therapeutic-dose anticoagulation in selected patients with thrombotic defects detected on postoperative CE-CT, as summarized in Supplementary Table 2. Antimicrobial prophylaxis did not differ across centers and followed standard institutional transplant protocols.

### Statistical analysis

Categorical variables are reported as absolute numbers and percentages and were compared using the χ^2^ test or Fisher’s exact test, as appropriate. Continuous variables are reported as median with interquartile range (IQR); normality was assessed using the Kolmogorov-Smirnov test. Group comparisons were performed using Student’s t test or Mann-Whitney U test, as appropriate.

Univariable and multivariable logistic regression analyses were performed to identify determinants of severe postoperative complications, prolonged LOS, and PTAP. LOS was primarily analyzed as a binary outcome using the third quartile as the threshold for prolonged hospitalization. As a sensitivity analysis, LOS was also analyzed as a continuous outcome using multivariable linear regression after logarithmic transformation to account for its right-skewed distribution. Logistic regression was selected for early postoperative outcomes occurring within a fixed 30-day window. Results are reported as odds ratios (OR) with 95% confidence intervals (CIs). Multivariable models were constructed by entering variables significantly associated with the outcome on univariable analysis, followed by backward elimination for covariate selection. Center-specific effects were included in multivariable models to account for between-center heterogeneity. The number of covariates included in each model was limited according to the number of outcome events, aiming for up to 10 outcome events per covariate, to reduce the risk of overfitting. Missing data were not imputed; multivariable analyses were performed as complete-case analyses for the variables included in each model. Variables with low event counts were interpreted cautiously because of the potential for unstable estimates. To address the potential impact of non-systematic imaging, a sensitivity analysis restricted to patients who underwent postoperative CE-CT was performed for severe postoperative complications and prolonged LOS.

Death-censored pancreas GS and patient survival were estimated using the Kaplan-Meier method and compared using the log-rank test. Predictors of death-censored graft loss were assessed using a Fine-Gray competing-risk regression [11], with death with a functioning graft treated as a competing event. Results are reported as subdistribution hazard ratios (sHRs) with 95% CIs.

Receiver operating characteristic (ROC) curve analysis was used to assess the discriminatory performance of serum amylase levels for predicting radiological GP. Optimal cut-off values were determined using the Youden index.

All tests were 2-sided, and *P* < 0.05 was considered statistically significant. Analyses were performed using SPSS software version 29.0.2 for Mac (IBM Corp., Armonk, NY, USA), GraphPad Prism version 10.2.2 for Mac (GraphPad Software, Boston, MA, USA) and R version 4.4.2 (R Foundation for Statistical Computing, Vienna, Austria) through RStudio version 2024.12.1 + 563 (RStudio, Boston, MA, USA), using the “survival” and “cmprsk” packages.

## Results

### Study population

A total of 453 consecutive patients undergoing PT were screened across the three participating centers. Twenty-one patients were excluded because of missing critical data required to apply the PTAP definition, namely unavailable serum pancreatic amylase measurements on POD1/POD2. The study cohort therefore included 432 PT recipients. Among these, 16 patients underwent graft pancreatectomy on POD0-1 because of early graft thrombosis and were excluded from the PTAP analysis because sustained POH over 48 h could not be assessed. The final analyzable cohort included 416 patients. All analyses assessing PTAP and its association with postoperative and long-term outcomes were performed in this cohort. Sustained postoperative hyperamylasemia (POH) occurred in 196 patients (47%), and PTAP was diagnosed in 86 (21%). Overall, postoperative CE-CT was performed in 371/416 patients (89%) in the analyzable cohort. Center-level CE-CT rates were 97/128 (76%) in the center performing CE-CT according to clinical indication, 138/152 (91%) in the center where protocol-driven CE-CT was implemented from 2013 onward, and 136/136 (100%) in the center performing routine protocol-driven CE-CT throughout the study period. Center-level PTAP rates were 38/128 (30%), 32/152 (21%), and 16/136 (12%), respectively (P = 0.002). PTAP rates were stable across transplant eras: 15/84 patients (18%) in 2010–2014, 29/141 patients (21%) in 2015–2019, and 42/191 patients (22%) in 2020–2024 (*P* = 0.738).

Among the remaining patients with sustained POH (n = 110), 85 had no radiological evidence of GP, whereas 25 did not undergo CE-CT. The latter patients were classified as having POH without radiological confirmation. Among these patients, 9 experienced a major postoperative complication, including 7 hemorrhagic and 2 thrombotic events. Among patients without sustained POH (n = 220), CE-CT was performed postoperatively in 200 (91%), and radiological findings consistent with GP were identified in 58 (29%). The study flow diagram is shown in Figure 1.

### Comparison of clinical characteristics according to PTAP status

Recipient and donor baseline characteristics were largely comparable between patients with and without PTAP (Table 1). In contrast, operative and preservation-related variables differed between groups. Median CIT was significantly longer in patients with PTAP than in those without PTAP [538 min (IQR, 458–630) vs. 489 min (IQR, 410–560); *P* < 0.001]. Median anastomotic time was also longer in the PTAP group [35 min (IQR, 30–40) vs. 30 min (IQR, 27–36), *P* = 0.036], as was overall duration of surgery (*P* = 0.022).

**TABLE 1:** Comparison of recipient and donor characteristics, surgical details and postoperative outcomes between patients with and without post-transplant acute pancreatitis (PTAP).

Variable	Overall *n* = 416 (%)	No PTAP *n* = 330 (%)	PTAP *n* = 86 (%)	p
Recipient features				
Sex, male	231 (56)	178 (54)	53 (62)	0.201
Age at transplant, years^a^	41 (35–47)	41 (34–47)	43 (38–48)	0.059
BMI, Kg/m^2a^	22.8 (20.9–25.5)	23 (21–25.5)	22.6 (20.6–25.7)	0.592
Dialysis	292 (71)	233 (71)	59 (70)	0.947
Diabetes type Type I Other	406 (98) 10 (2)	322 (98) 8 (2)	84 (98) 2 (2)	1.000
Previous pancreas transplant	13 (3)	12 (4)	1 (1)	0.240
Donor features				
Donor type, DBD	407 (98)	321 (97)	86 (100)	0.214
Sex, male	252 (62)	204 (63)	48 (57)	0.328
Age, years^a^	30 (22–38)	30 (22–38)	30 (22–40)	0.584
BMI, Kg/m^2a^	23.4 (21–25.4)	23.5 (21–25.4)	23.4 (21.2–25.7)	0.573
Cold ischemia time, minutes^a^ ^,^ ^b^	496 (420–578)	489 (410–560)	538 (458–630)	**<0.001**
Anastomotic time, minutes^a^ ^,^ ^b^	30 (28–40)	30 (27–36)	35 (30–40)	**0.036**
Intraoperative details				
Transplant type SPK PAK PTA	362 (87) 13 (3) 41 (10)	283 (86) 13 (4) 34 (10)	79 (92) 0 (0) 7 (8)	0.135
Duration of surgery, minutes^a^	290 (240–330)	285 (240–322)	304 (257–341)	**0.022**
Intraoperative transfusions	78 (19)	60 (19)	18 (21)	0.579
Postoperative outcomes				
Postoperative complications, CD ≥ IIIA [10]	112 (27)	79 (24)	33 (38)	**0.008**
Length of hospital stay, days^a^	16 (13–23)	15 (13–21)	20 (14–33)	**<0.001**
Early relaparotomy^c^	101 (24)	71 (22)	30 (35)	**0.010**
Early hemorrhage^c^	88 (21)	65 (20)	23 (27)	0.154
Early thrombosis^c^	83 (20)	60 (18)	23 (27)	0.077
Early graft explantation (POD 2–30)	26 (6)	13 (4)	13 (15)	**<0.001**
Duodenal leakage	13 (3)	6 (2)	7 (8)	**0.007**
Graft rejection (before discharge)^b^	10 (3)	8 (3)	2 (2)	1.000

*BMI*, body mass index; *CD*, Clavien-Dindo; *DBD*, deceased after brain death; *PAK*, pancreas after kidney; *POD,* postoperative day; *PTA*, pancreas transplant alone; *SPK*, simultaneous pancreas and kidney. Bold values indicate statistical significance.

^a^ Expressed as median (IQR).

^b^ Missing data: CIT n = 12, anastomotic time n = 169, graft rejection before discharge n = 10.

^c^ Complications defined as early when occurring within 30 days after surgery.

With respect to early postoperative outcomes, graft explantation between POD2 and POD30 was more frequent in patients with PTAP than in those without PTAP [13/86 (15%) vs. 13/330 (4%), *P* < 0.001]. Duodenal leakage was also more common in the PTAP group [7/86 (8%) vs. 6/330 (2%), *P* = 0.007], whereas rates of early hemorrhage and thrombosis did not differ significantly between groups. Among the 13 patients with duodenal leakage, 11 had sustained POH and 7 fulfilled the adapted ISGPS-based PTAP definition.

### Primary outcomes

Severe postoperative complications (Clavien-Dindo grade ≥ IIIA) occurred in 112 patients (27%) and were significantly more frequent in patients with PTAP than in those without PTAP [33/86 (38%) vs. 79/330 (24%); *P* = 0.008]. Univariable and multivariable logistic regression analyses assessing determinants of severe postoperative complications are reported in Table 2. After adjustment for center-specific effects and clinically relevant covariates, PTAP was the only independent predictor of severe postoperative complications (OR, 2.482; 95% CI, 1.432–4.305; *P* = 0.001).

**TABLE 2:** Univariable and multivariable analyses assessing the determinants of severe postoperative complications (n = 404).

​	*Univariable*			*Multivariable* ^a^		
Variable	OR	95% CI	*p*	OR	95% CI	*p*
Recipient sex, female	0.865	0.558–1.340	0.515	​	​	​
Recipient age, years	0.983	0.957–1.009	0.188	​	​	​
Recipient BMI, kg/m^2^	0.996	0.939–1.056	0.885	​	​	​
Recipient diabetes type, type I	0.859	0.218–3.382	0.828	​	​	​
Recipient on dialysis	1.094	0.675–1.772	0.716	​	​	​
Donor sex, female	0.904	0.576–1.418	0.660	​	​	​
Donor age, years	1.002	0.981–1.024	0.863	​	​	​
Donor BMI, kg/m^2^	1.044	0.970–1.123	0.252	​	​	​
Donor type, DCD	1.362	0.335–5.542	0.666	​	​	​
Cold ischemia time, minutes	0.998	0.996–1.000	**0.049**	–	–	–
Anastomotic time, minutes^b^	1.014	0.992–1.036	0.221	​	​	​
Transplant type, PAK/PTA	1.327	0.712–2.473	0.373	​	​	​
Previous PT	0.899	0.239–3.383	0.875	​	​	​
Intraoperative transfusions	0.541	0.290–1.011	0.054	​	​	​
PTAP	1.970	1.192–3.258	**0.008**	2.482	1.432–4.305	**0.001**

*BMI*, body mass index; *CI*, confidence interval; *DCD*, deceased after circulatory death; *OR*, odds ratio; *PAK*, pancreas after kidney; *PT,* pancreas transplant; *PTA*, pancreas transplant alone; *PTAP*, post-transplant acute pancreatitis. Bold values indicate statistical significance.

^a^ Multivariable analysis adjusted for center.

^b^ Available for *n* = 247 patients.

Median LOS was significantly longer in patients with PTAP than in the remainder of the cohort (20 vs. 15 days, *P* < 0.001). On multivariable analysis (Table 3), PTAP (OR, 3.817; 95% CI, 2.146–6.792, *P* < 0.001) and graft rejection before discharge (OR, 16.662; 95% CI, 3.063–90.621; *P* = 0.001) were independently associated with prolonged LOS (>23 days–*n* = 98). PTAP remained independently associated with longer hospital stay when LOS was analyzed as a continuous log-transformed outcome (adjusted exp[β], 1.301; 95% CI, 1.166–1.451; P < 0.001 – Supplementary Table 3).

**TABLE 3:** Univariable and multivariable analyses assessing the determinants of prolonged length of hospital stay (>23 days) (n = 395).

​	*Univariable*			*Multivariable* ^a^		
Variable	OR	95% CI	*p*	OR	95% CI	*p*
Recipient sex, female	0.813	0.514–1.288	0.378	​	​	​
Recipient age, years	0.981	0.954–1.008	0.167	​	​	​
Recipient BMI, kg/m^2^	0.974	0.915–1.036	0.399	​	​	​
Recipient diabetes type, type I	0.717	0.182–2.828	0.635	​	​	​
Recipient on dialysis	1.281	0.766–2.143	0.344	​	​	​
Donor sex, female	1.294	0.815–2.055	0.274	​	​	​
Donor age, years	1.019	0.996–1.042	0.104	​	​	​
Donor BMI, kg/m^2^	1.014	0.940–1.094	0.714	​	​	​
Donor type, DCD	2.647	0.697–10.057	0.153	​	​	​
Cold ischemia time, minutes	0.998	0.996–1.000	**0.044**	–	–	–
Anastomotic time, minutes^b^	1.005	0.989–1.021	0.560	​	​	​
Transplant type, PAK/PTA	0.522	0.237–1.147	0.106	​	​	​
Previous PT	0.966	0.261–3.582	0.959	​	​	​
Intraoperative transfusions	0.549	0.283–1.067	0.549	​	​	​
PTAP	2.887	1.733–4.809	**<0.001**	3.817	2.146–6.792	**<0.001**
Graft rejection (before discharge)	13.511	2.819–64.764	**0.001**	16.662	3.063–90.621	**0.001**

*BMI*, body mass index; *CI*, confidence interval; *DCD*, deceased after circulatory death; *OR*, odds ratio; *PAK*, pancreas after kidney; *PT,* pancreas transplant; *PTA*, pancreas transplant alone; *PTAP*, post-transplant acute pancreatitis. Bold values indicate statistical significance.

^a^ Multivariable analysis adjusted for center.

^b^ Available for *n* = 247 patients.

In a sensitivity analysis restricted to the 371 patients who underwent postoperative CE-CT, severe postoperative complications occurred in 33/86 patients with PTAP (38%) and in 67/285 patients without PTAP (23%; *P* = 0.008). Median hospital stay was longer in patients with PTAP than in those without PTAP (20 days [IQR, 14–33] vs. 15 days [IQR, 13–22]; *P* < 0.001). On multivariable analysis, PTAP remained independently associated with severe postoperative complications (OR, 2.362; 95% CI, 1.369–4.077; *P* = 0.002) and prolonged hospital stay (OR, 3.767; 95% CI, 2.140–6.632; *P* < 0.001).

### Outcomes according to biochemical and radiological PTAP components

To further characterize the relative contribution of sustained POH and radiological GP to postoperative outcomes, a four-group comparison was performed among patients who underwent postoperative CE-CT. Overall, 142 (38%) patients had neither sustained POH nor radiological GP, 85 (23%) had sustained POH without radiological GP, 58 (16%) had radiological GP without sustained POH, and 86 (23%) fulfilled the PTAP definition. CE-CT timing was comparable across groups (*P* = 0.442). Later serum pancreatic amylase trajectories (POD3, POD5, and POD10), normalized to center-specific ULN, differed significantly across groups (*P* < 0.001), with the highest median values observed in patients with sustained POH, either with or without radiological GP. Patients with radiological GP only did not show a delayed median enzyme rise above the ULN.

Patients with radiological GP without sustained POH showed intermediate outcomes, with rates of severe postoperative complications (26%) and hospital stay (median 18 days; IQR, 13–28) closer to those observed in the no POH/no radiological GP and POH-only groups than to those observed in patients fulfilling the combined PTAP definition (severe complications, 38%; median LOS, 20 days). The full four-group comparison is reported in Supplementary Table 4.

### Long-term outcomes

**FIGURE 2:**
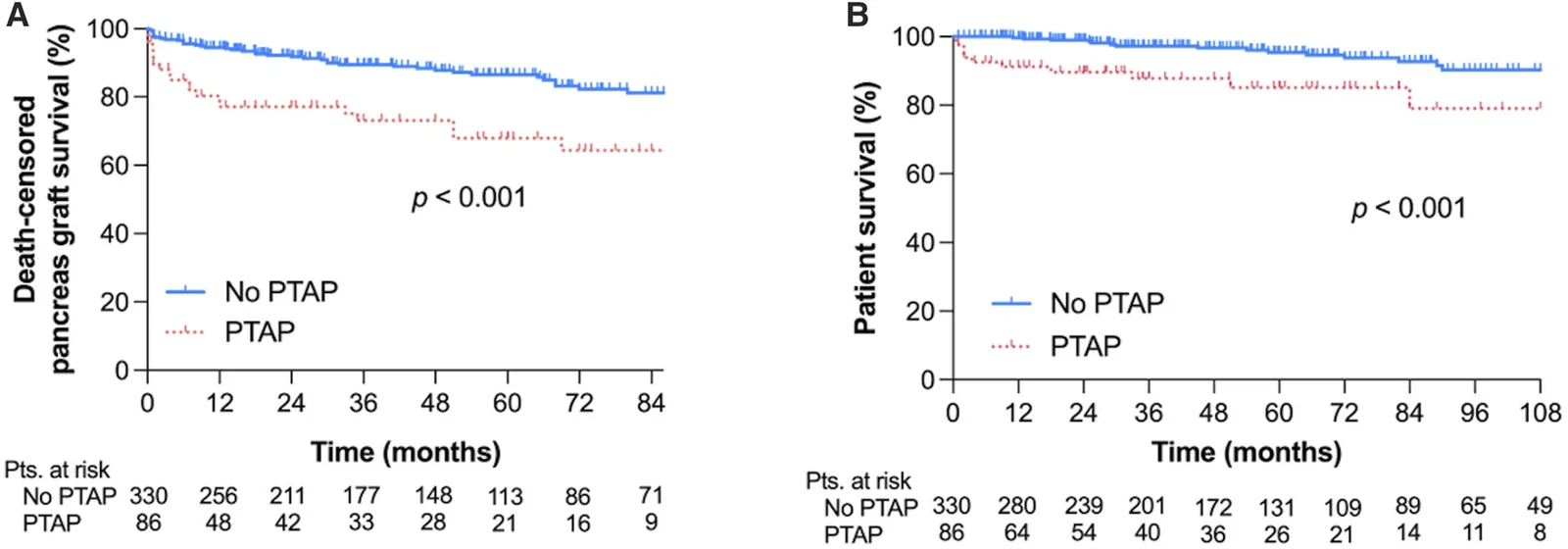
Kaplan-Meier curves comparing death-censored pancreas graft survival **(A)** and patient survival **(B)** between patients with and without post-transplant acute pancreatitis (PTAP). Survival curves were compared using the log-rank test.

After a median follow-up of 51 months (IQR, 24–87), graft loss occurred in 85 patients (20%), and 30 patients (7%) died. Death-censored pancreas GS was significantly worse in patients with PTAP than in those without PTAP (*P* < 0.001). Three-year death-censored pancreas graft survival was 86% in patients without PTAP and 65% in patients with PTAP (Figure 2A). Overall pancreas graft rejection during follow-up occurred in 58/416 patients (14%).

Results of univariable and multivariable competing-risk regression analyses for predictors of death-censored pancreas graft loss are reported in Table 4. On multivariable analysis, independent predictors of pancreas graft loss included donor type (DCD; sHR, 6.586; 95% CI, 1.409–30.774; *P* = 0.017), CIT (sHR, 1.004 per minute; 95% CI, 1.001–1.006; *P* = 0.003), transplant type (PAK/PTA; sHR, 2.722; 95% CI, 1.314–5.641; *P* = 0.007), PTAP (sHR, 3.412; 95% CI, 2.005–5.808; *P* < 0.001), early thrombosis (sHR, 2.821; 95% CI, 1.626–4.895; *P* < 0.001), and graft rejection (sHR, 3.975; 95% CI, 2.338–6.616; *P* < 0.001).

**TABLE 4:** Univariable and multivariable Fine-Gray competing risks regression analyses assessing the determinants of death-censored pancreas graft survival after pancreas transplantation (n = 395).

​	*Univariable*			*Multivariable* ^a^		
Variable	sHR	95% CI	*p*	sHR	95% CI	*p*
Recipient sex, female	1.042	0.683–1.591	0.848	​	​	​
Recipient age, years	1.006	0.984–1.030	0.581	–	–	–
Recipient BMI, kg/m^2^	0.990	0.936–1.049	0.743	​	​	​
Recipient diabetes type, type I	2.240	0.294–17.055	0.436	​	​	​
Recipient on dialysis^b^	0.583	0.394–0.863	**0.007**	​	​	​
Donor type, DCD	1.759	0.441–7.023	0.424	6.586	1.409–30.774	**0.017**
Donor sex, female	0.993	0.638–1.544	0.974	​	​	​
Donor age, years	0.989	0.966–1.012	0.343	–	–	–
Donor BMI, kg/m^2^	0.949	0.876–1.029	0.207	​	​	​
Cold ischemia time, minutes	1.002	1.001–1.004	**0.004**	1.004	1.001–1.006	**0.003**
Anastomotic time, minutes^c^	1.011	0.996–1.025	0.153	​	​	​
Intraoperative transfusions	0.992	0.593–1.659	0.976	​	​	​
Transplant type, PAK/PTA	2.791	1.780–4.378	**<0.001**	2.722	1.314–5.641	**0.007**
Previous PT	0.951	0.280–3.232	0.936	​	​	​
PTAP	2.580	1.654–4.025	**<0.001**	3.412	2.005–5.808	**<0.001**
Early thrombosis^d^	2.904	1.850–4.558	**<0.001**	2.821	1.626–4.895	**<0.001**
Early hemorrhage^d^	1.021	0.597–1.746	0.939	​	​	​
Rejection	3.552	2.305–5.473	**<0.001**	3.975	2.338–6.616	**<0.001**

*BMI*, body mass index; *CI*, confidence interval; *DCD*, deceased after circulatory death; s*HR*, subdistribution hazard ratio; *PAK*, pancreas after kidney; *PT,* pancreas transplant; *PTA*, pancreas transplant alone; *PTAP*, post-transplant acute pancreatitis. Bold values indicate statistical significance.

^a^ Multivariable analysis adjusted for center.

^b^ Not included in the multivariable model due to collinearity with transplant type.

^c^ Available for *n* = 247 patients.

^d^ Complications defined as early when occurring within 30 days after surgery.

To further characterize death-censored pancreas graft loss, timing, associated graft-related events, and main causes of graft loss were assessed according to PTAP status (Supplementary Table 5). Among patients experiencing graft loss (*n* = 85), median time to graft loss was shorter in patients with PTAP than in those without PTAP (1.0 months [IQR, 0–7.5] vs. 9.3 months [IQR, 1–41]; *P* = 0.015). Early graft explantation between POD2 and POD30 was more frequent among patients with PTAP (13/31, 42%) than among those without PTAP (13/54, 24%), although this difference did not reach statistical significance (*P* = 0.085). Pancreas graft rejection before discharge occurred in 10/416 patients (2%), while overall pancreas graft rejection during follow-up occurred in 58/416 patients (14%). The distribution of graft-loss causes differed according to PTAP status (*P* = 0.036), with early postoperative complication-related graft loss accounting for a larger proportion of events among patients with PTAP, whereas rejection-related graft loss was more common among patients without PTAP.

To assess whether the association between PTAP and graft loss was driven by very early technical failures, a sensitivity analysis was performed after excluding patients who underwent early graft explantation between POD2 and POD30. In Kaplan-Meier analysis, death-censored pancreas graft survival remained significantly worse in patients with PTAP (*P* = 0.003). In a center-adjusted competing-risk sensitivity analysis, PTAP remained associated with a higher cumulative incidence of pancreas graft loss (sHR, 1.964; 95% CI, 1.087–3.550; *P* = 0.025).

Patient survival was also significantly worse among patients with PTAP (*P* < 0.001, Figure 2B).

### Radiological graft pancreatitis and predictors of PTAP

Overall, 371 patients (89%) underwent at least one CE-CT scan during the postoperative period, at a median of POD8 (IQR, 4–10). Among these patients, 171 (45%) had sustained POH. Radiological signs consistent with GP were identified in 144 patients (35%), of whom 86 met the biochemical criterion for sustained POH and were therefore classified as having PTAP.

**FIGURE 3:**
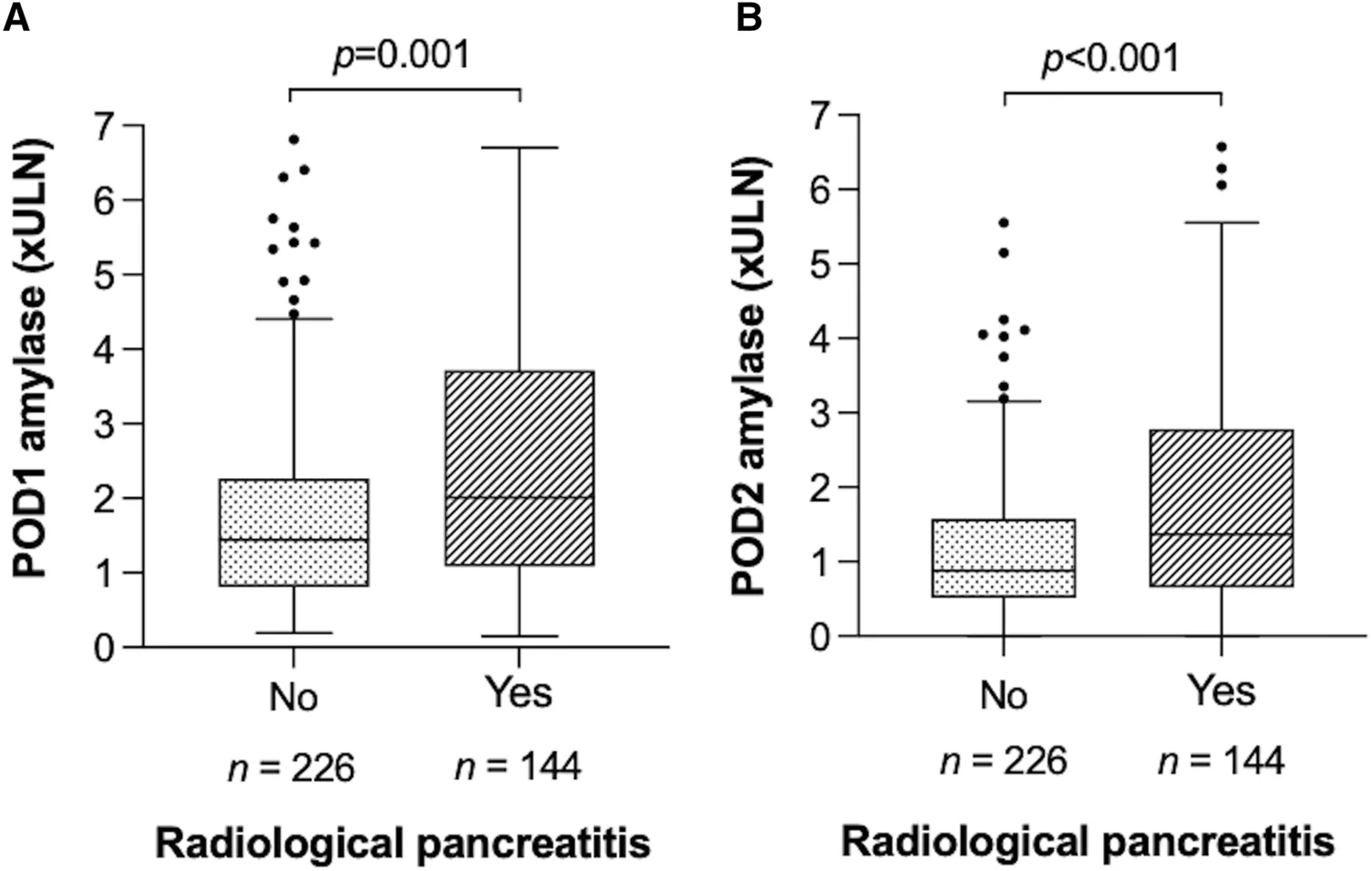
Comparison of serum amylase levels on postoperative day (POD) 1 **(A)** and POD 2 **(B)**, expressed as multiples of the laboratory upper limit of normal (ULN), between patients with and without radiological evidence of graft pancreatitis. Box plots represent median and interquartile range, with whiskers according to Tukey (1.5×IQR). Black dots indicate outliers.

Serum amylase levels on POD1 and POD2, expressed as multiples of the upper limit of normal (ULN), were significantly higher in patients with radiological GP than in the remainder of the cohort (*P* < 0.001, Figure 3). The discriminatory performance of POD1 and POD2 amylase levels for predicting radiological GP was modest, with an AUC of 0.630 (95% CI, 0.570–0.690; *P* < 0.001) and 0.648 (95% CI, 0.589–0.708; *P* < 0.001), respectively (Supplementary Figure 1). Optimal cut-off values were 3.25×ULN on POD1 and 2.25×ULN on POD2. Radiological GP was present in 74% of patients (37/50) who exceeded both these thresholds, compared with 33% of those below at least one threshold (*P* < 0.001).

Determinants of PTAP were further explored using logistic regression analyses (Supplementary Table 6). On multivariable analysis, CIT was the only independent determinant of PTAP (OR, 1.004 per minute; 95% CI 1.002–1.006; *P* < 0.001).

## Discussion

In this multicenter study, the ISGPS definition of PPAP [7] was adapted to the setting of pancreas transplantation to define post-transplant acute pancreatitis (PTAP) and assess its clinical relevance in a large cohort of PT recipients. PTAP was identified in approximately 1 in 5 patients and was consistently associated with increased postoperative morbidity, prolonged hospitalization, and worse long-term graft survival. These findings suggest that PTAP represents a clinically meaningful manifestation of early graft injury rather than a benign biochemical epiphenomenon. Importantly, applying the ISGPS framework to the transplant setting does not imply shared pathophysiological mechanisms between PTAP and PPAP, but rather the use of a standardized, outcome-oriented approach to identify clinically relevant perioperative pancreatic graft injury.

Although the present study does not support specific changes in perioperative management, the findings may have important implications for clinical decision-making after PT. Identification of PTAP using objective biochemical and radiological criteria may enable early risk stratification of patients at increased risk for major postoperative complications and graft loss. In this context, PTAP may be viewed as a warning signal prompting closer clinical surveillance and timely imaging, while potentially informing individualized postoperative drain management rather than serving as a trigger for predefined therapeutic interventions.

GP after PT has long been recognized as a relevant complication, but its reported incidence and clinical significance have varied markedly across studies. This variability largely reflects the absence of a standardized and validated definition [5, 12], with previous reports relying on heterogeneous combinations of clinical symptoms, enzyme elevations, imaging findings, and pathological features [4, 13–16]. As highlighted by a recent scoping review [5], reported rates of GP range from 0% to 87%, reflecting both true inter-center variability (i.e. differences in donor characteristics, CIT, operative complexity, and perioperative factors) and the lack of a universally accepted definition. This heterogeneity has limited comparability across centers and hindered both clinical decision-making and research in the field. Established definitions of native acute pancreatitis are not directly applicable to the transplant setting because of the unique perioperative and immunological milieu of PT. Early graft injury is strongly influenced by ischemia-reperfusion damage [17, 18], microvascular dysfunction, denervation, and immunosuppression-related factors, all of which may lead to pancreatic enzyme release without overt inflammatory sequelae. Accordingly, POH is common after PT and has often been regarded as a benign biochemical finding [17, 19]. However, increasing evidence suggests that persistent enzyme elevation may identify a subset of patients at increased risk of clinically relevant complications [15, 20–22].

In pancreatic surgery, the ISGPS has recently proposed and validated a standardized definition of PPAP [7, 8] based on sustained POH and radiological evidence of pancreatitis. This framework was developed to distinguish clinically relevant pancreatitis from isolated biochemical abnormalities and has shown prognostic significance in surgical cohorts. Although PPAP arises in a different clinical context, its conceptual structure, based on objective biochemical and imaging criteria, appears well suited to the perioperative phase of PT. In the present study, this framework was adapted to the transplant setting by applying these two objective components [7] while intentionally excluding the clinical management criterion. This choice was made to avoid incorporation bias, because changes in postoperative management are intrinsically linked to short-term outcomes, which were primary study endpoints. By relying exclusively on biochemical and radiological criteria, we sought to preserve the independence between exposure and outcome assessment.

Using this adapted framework, PTAP was diagnosed in 21% of patients. PTAP was the only independent predictor of severe postoperative complications and was strongly associated with prolonged length of stay. These findings indicate that PTAP identifies a subgroup of patients with clinically significant early graft injury and tangible consequences for postoperative recovery and resource utilization. PTAP was also independently associated with death-censored graft loss, with early separation of survival curves after transplantation. Although the association between PTAP and death-censored pancreas graft survival persisted after excluding very early graft failures requiring graft explantation between POD2 and POD30, this finding should be interpreted as prognostic rather than causal. Indeed, PTAP and other early postoperative complications, including thrombosis, rejection and graft exocrine leakage, may occur within the same postoperative trajectory, and their temporal and biological relationships cannot be fully disentangled in a retrospective study. Taken together, these observations suggest that PTAP likely captures a severe phenotype of early graft injury associated with a higher subsequent risk of graft failure, rather than a transient inflammatory process without longer-term implications.

Postoperative imaging played a central role in confirming the diagnosis of PTAP. In this cohort, CE-CT was performed in most patients on the basis of clinical indication rather than systematically. Although this approach may have led to underdiagnosis of mild or subclinical PTAP, it reflects real-world clinical practice and likely enriched the analysis for clinically meaningful cases. Interpretation of early postoperative imaging remains challenging, as inflammatory changes may overlap with expected postoperative findings or coexist with other graft-related complications, including thrombosis and rejection. Nevertheless, in the presence of sustained POH, radiological findings such as graft edema or peripancreatic fluid collections should raise suspicion for PTAP and prompt closer clinical surveillance [23].

Serum amylase levels on postoperative days 1 and 2 were significantly higher in patients with radiological GP, supporting their role as an early risk signal. However, the modest discriminatory performance observed in ROC analyses indicates that amylase levels alone should not be considered diagnostic for PTAP. Rather, persistent enzyme elevation may serve as an early warning marker to identify patients who may benefit from targeted imaging and closer monitoring during the postoperative course. It should also be acknowledged that POH may arise from causes other than GP, including duodenal leakage, although this complication was uncommon in the present cohort.

Although the etiological context of GP after PT differs from that of post-pancreatectomy acute pancreatitis, key downstream features, including parenchymal edema, inflammatory activation, and microcirculatory impairment, are conceptually translatable across settings. The four-group comparison highlighted the clinical relevance of combining biochemical and radiological criteria: patients fulfilling the PTAP definition had the highest burden of postoperative morbidity and the longest hospital stay, whereas those with either sustained POH alone or radiological GP alone showed a less pronounced clinical impact. Radiological abnormalities in the absence of sustained POD1-2 hyperamylasemia may therefore identify a different, less clinically severe phenotype. Consistently, postoperative enzyme trajectories further showed that patients with sustained POH had median serum pancreatic amylase values above the center-specific ULN up to POD10, whereas patients without sustained POH, including those with radiological GP alone, did not show a delayed median enzyme rise above the ULN. This suggests that sustained early POH may represent an early marker of clinically relevant graft injury rather than a merely transient postoperative enzyme elevation. Overall, the adapted ISGPS-based framework may represent a candidate operational definition for clinically meaningful GP after PT.

Identification of potentially modifiable risk factors for PTAP is of particular clinical interest. In the present study, prolonged CIT emerged as the only independent determinant of PTAP among the variables uniformly available for analysis. Although the OR per minute appears numerically small, the cumulative effect over prolonged ischemic intervals is likely to be clinically meaningful. This finding is consistent with a potential role of ischemia-reperfusion injury in the pathogenesis of PTAP and with experimental and clinical evidence showing impaired graft microcirculation, inflammatory activation, and tissue edema after prolonged cold preservation [24]. However, this association should be interpreted cautiously. CIT likely reflects not only cold ischemic injury itself, but also preservation-related, procurement-related, and logistical factors. Data on additional donor characteristics, procurement distance, detailed transport logistics, and pancreas procurement time were not available with sufficient completeness across centers, and residual confounding from these factors cannot be excluded. Future prospective studies should clarify whether strategies aimed at reducing ischemia-preservation burden can decrease the risk of PTAP.

Several limitations should be acknowledged. First, the retrospective design introduces the potential for selection and information bias. Second, patients requiring graft pancreatectomy within the first postoperative day were necessarily excluded because sustained hyperamylasemia could not be assessed. As these cases may represent the most severe end of the early GP spectrum, including ischemia-reperfusion-related pancreatitis, their exclusion may have led to underestimation of the incidence of severe early GP after PT. However, retrospective pathological distinction between ischemia-reperfusion-related pancreatitis and thrombosis-related ischemic injury would be unreliable. Third, postoperative imaging was not performed systematically in all patients, which may have resulted in underdiagnosis of mild disease and differential ascertainment across centers and time periods. In particular, patients with sustained POH who did not undergo CE-CT could not be definitively classified with respect to radiological GP and may have included mild or subclinical cases. However, CE-CT was performed in most patients, and the association between PTAP and the main postoperative outcomes remained consistent in a sensitivity analysis restricted to patients who underwent postoperative CE-CT. In addition, postoperative CE-CT scans were interpreted locally as part of routine clinical care, and no central blinded radiological review was performed. Therefore, prospective validation using standardized imaging protocols is warranted. Fourth, radiological findings consistent with pancreatitis may overlap with other early graft complications, such as thrombosis, acute graft rejection, or graft exocrine leakage, and causal relationships between PTAP and associated outcomes cannot be definitively established. We also did not retrospectively classify clinically relevant management changes as attributable to PTAP. In the early postoperative course after PT, management decisions are frequently driven by overlapping complications. Because no standardized definition of GP was available at the time of clinical management, attributing specific therapeutic decisions retrospectively to PTAP would have been prone to misclassification and circular reasoning. For this reason, the adapted definition was intentionally restricted to objective biochemical and radiological criteria. In addition, serum lipase levels, complete enzyme kinetics beyond the first 48 postoperative hours, and drain amylase measurements were not systematically available across centers. Finally, perioperative management strategies, including anticoagulation protocols, varied across centers and over time. PTAP rates were stable across transplant eras, but center-level differences were observed and may reflect differences in surgical technique, imaging strategies, postoperative management, case mix, and unmeasured recipient-related factors, including vascular complexity. Although adjustment for center-specific effects was performed to mitigate the impact of this heterogeneity, residual confounding related to center-specific management strategies cannot be excluded.

In conclusion, an adapted ISGPS-based definition identified PTAP as a frequent and clinically meaningful complication after PT. PTAP identifies a subgroup of patients with clinically relevant early graft injury and was associated with major postoperative complications, prolonged hospitalization, and impaired pancreas graft survival, supporting its role as a prognostic marker rather than establishing a direct causal relationship. Standardized diagnostic criteria may improve recognition of clinically meaningful GP, support closer clinical surveillance and early imaging, facilitate comparisons across transplant programs, and provide a foundation for prospective studies aimed at refining diagnostic thresholds and informing postoperative management. Given the retrospective design, non-systematic imaging, and potential overlap with other early graft-related complications, prospective validation using standardized imaging protocols, prespecified CT timing and central radiological adjudication is warranted.

## Data Availability

The raw data supporting the conclusions of this article will be made available by the corresponding author upon reasonable request, in anonymized form and in accordance with applicable privacy and ethical regulations.
