# Supplementary material for: Post-transplant acute pancreatitis after pancreas transplantation: application of the International Study Group for Pancreatic Surgery definition and clinical impact in a multicenter cohort study
Source: Transpl Int. 2026 Sep 8;39:16849. doi: 10.3389/ti.2026.16849 (PMC13593580; doi:10.3389/ti.2026.16849)
Supplement: Supplementary file 1 [file Table1.docx]

**Supplementary Table 1**. Institutional upper limits of normal (ULN) per center of serum amylase.

| **Center** | **Serum amylase ULN** |
| --- | --- |
| Center 1 | 100 U/L |
| Center 2 | 53 U/L |
| Center 3 | 125 U/L |

*ULN*, upper limit of normal

**Supplementary Table 2.** Center-specific anticoagulation protocols.

| **Center** | **Anticoagulation regimen** |
| --- | --- |
| **Center 1** | Prophylactic-dose LMWH was administered for 30 days after transplantation.  Therapeutic-dose anticoagulation was introduced in case of thrombotic defects detected on postoperative CE-CT. |
| **Center 2** | Continuous intravenous UFH at 300 IU/h was administered until recovery of renal graft function, followed by prophylactic-dose LMWH for 30 days after transplantation. Therapeutic-dose anticoagulation was introduced in case of thrombotic defects detected on postoperative CE-CT and continued at least until the following CE-CT. |
| **Center 3** | Until 2018, therapeutic-dose LMWH was routinely administered after transplantation.  From 2018 onward, prophylactic-dose LMWH was administered, unless thrombotic defects were detected on postoperative CE-CT, in which case therapeutic-dose LMWH was introduced. |
| *CE-CT*, contrast-enhanced computed tomography; *LMWH*, low-molecular-weight heparin; *UFH*, unfractionated heparin. | |

| **Variable** | **Adjusted exp( β)** | **95% CI** | **p** |
| --- | --- | --- | --- |
| **Donor age**, years | ­– | – | **–** |
| **Donor type**, DCD | ­– | – | **–** |
| **Transplant type**, PAK/PTA | 0.849 | 0.729-0.989 | **0.036** |
| **PTAP** | 1.301 | 1.166-1.451 | **<0.001** |
| **Graft rejection** (before discharge) | 1.697 | 1.287-2.241 | **<0.001** |
| *DCD*, deceased after circulatory death; *PAK*, pancreas after kidney; *PTA*, pancreas transplant alone; *PTAP*, post-transplant acute pancreatitis. Bold values indicate statistical significance.  Bold values indicate statistical significance (P<0.05). | | | |

**Supplementary Table 3.** Center-adjusted multivariable analysis assessing the determinants of length of hospital stay as a continuous log-transformed outcome.

**Supplementary Table 4.** Four-group comparison according to sustained postoperative hyperamylasemia and radiological graft pancreatitis among patients undergoing postoperative CE-CT

| **Variable** | **No POH / no radiological GP (n=142)** | **POH only (n=85)** | **Radiological GP only (n=58)** | **PTAP (n=86)** | **p** |
| --- | --- | --- | --- | --- | --- |
| **CE-CT timing**, POD | 7 (4-10) | 8 (4-10) | 9 (5-11) | 8 (4-10) | 0.442 |
| **POD 1 amylase**, ×ULN (*n*=371) | 0.91 (0.62-1.46) | 2.57 (1.98-4.00) | 1.02 (0.62-1.51) | 3.57 (2.20-5.24) | **<0.001** |
| **POD 2 amylase**, ×ULN (*n*=371) | 0.57 (0.40-0.84) | 1.68 (1.27-2.39) | 0.60 (0.37-0.77) | 2.56 (1.66-3.94) | **<0.001** |
| **POD 3 amylase**, ×ULN (*n*=358) | 0.55 (0.36-0.75) | 1.11 (0.77-1.54) | 0.47 (0.29-0.66) | 1.13 (0.84-2.72) | **<0.001** |
| **POD 5 amylase**, ×ULN (*n*=347) | 0.76 (0.51-1.29) | 1.15 (0.77-1.93) | 0.62 (0.47-0.98) | 1.03 (0.65-1.70) | **<0.001** |
| **POD 10 amylase**, ×ULN (*n*=310) | 0.86 (0.51-1.36) | 1.25 (0.99-1.99) | 0.92 (0.54-1.34) | 1.18 (0.67-1.77) | **<0.001** |
| **Graft edema/peripancreatic fat stranding** | – | – | 28 (48) | 49 (57) | 0.305 |
| **(Peri)pancreatic fluid collections** | – | – | 39 (67) | 64 (74) | 0.349 |
| **Pancreatic/peripancreatic necrosis** | – | – | 4 (7) | 5 (6) | 1.000 |
| **Postoperative complications**, CD ≥IIIA | 31 (22) | 21 (25) | 15 (26) | 33 (38) | **0.049** |
| **Length of hospital stay**, days | 15 (13-20) | 16 (13-20) | 18 (13-28) | 20 (14-33) | **<0.001** |
| **Early relaparotomy**^a^ | 28 (20) | 20 (24) | 13 (22) | 30 (35) | 0.074 |
| **Early hemorrhage**^a^ | 26 (18) | 20 (24) | 12 (21) | 23 (27) | 0.487 |
| **Early thrombosis**^a^ | 21 (15) | 16 (19) | 15 (26) | 23 (27) | 0.106 |
| **Early graft explantation** (POD 2–30) | 2 (1) | 3 (4) | 1 (2) | 13 (15) | **<0.001** |
| **Duodenal leakage** | 1 (1) | 3 (4) | 1 (2) | 7 (8) | **0.015** |
| **Graft rejection** (before discharge) | 4 (3) | 2 (2) | 2 (3) | 2 (2) | 1.000 |
| **3-year death-censored pancreas graft survival rate**, % | 90 (84-95) | 85 (76-94) | 95 (89-100) | 65 (55-76) | **<0.001** |
| **3-year patient survival rate**, % | 97 (94-100) | 98 (95-100) | 98 (93-100) | 86 (78-94) | **0.001** |
| *CD*, Clavien-Dindo; *CE-CT*, contrast-enhanced computed tomography; *GP*, graft pancreatitis; *POD*, postoperative day; *POH*, postoperative hyperamylasemia; *PTAP*, post-transplant acute pancreatitis.  Categorical variables are reported as n (%), continuous variables are reported as median (IQR) or rate (95% CI), and survival estimates as rate (95% CI). ^a^Events occurring within 30 postoperative days.  Bold values indicate statistical significance (P<0.05). | | | | | |

**Supplementary Table 5.** Timing, associated events, and causes of death-censored pancreas graft loss according to PTAP status.

| **Variable** | **No PTAP (n=54)** | **PTAP (n=31)** | **p** |
| --- | --- | --- | --- |
| **Time to graft loss**, months | 9.3 (1.0-41.0) | 1.0 (0-7.5) | **0.015** |
| **Early graft explantation** (POD2-30) | 13 (24) | 13 (42) | 0.085 |
| **Duodenal leakage** | 2 (4) | 5 (16) | 0.094 |
| **Early thrombosis** | 20 (37) | 10 (32) | 0.657 |
| **Graft rejection before discharge** | 2 (4) | 0 (0) | 0.517 |
| **Overall graft rejection** | 23 (43) | 5 (16) | **0.012** |
| **Causes of graft loss**  Explantation within POD30^*^  Rejection  Other causes | 13 (24)  23 (43)  18 (33) | 13 (42)  5 (16)  13 (42) | **0.036** |
| Data are reported as n (%) or median (IQR). POD, postoperative day; PTAP, post-transplant acute pancreatitis.  *Explantation due to postoperative complications  Bold values indicate statistical significance (P<0.05). | | | |

**Supplementary Table 6.** Univariable and multivariable analyses assessing the determinants of post-transplant acute pancreatitis.

|  | ***Univariable*** | | | ***Multivariablea*** | | |
| --- | --- | --- | --- | --- | --- | --- |
| **Variable** | **OR** | **95% CI** | ***p*** | **OR** | **95% CI** | ***p*** |
| **Recipient sex,** female | 0.729 | 0.449-1.185 | 0.202 |  |  |  |
| **Recipient** **age**, years | 1.022 | 0.994-1.052 | 0.121 |  |  |  |
| **Recipient BMI,** kg/m^2^ | 0.994 | 0.932-1.061 | 0.861 |  |  |  |
| **Recipient diabetes type**, type I | 1.043 | 0.218-5.006 | 0.958 |  |  |  |
| **Recipient on dialysis** | 0.876 | 0.468-1.350 | 0.548 |  |  |  |
| **Donor sex,** female | 1.275 | 0.783-2.076 | 0.329 |  |  |  |
| **Donor** **age**, years | 1.009 | 0.985-1.033 | 0.480 |  |  |  |
| **Donor BMI**, kg/m^2^ | 1.031 | 0.951-1.118 | 0.452 |  |  |  |
| **Cold ischemia time**, minutes | 1.004 | 1.002-1.006 | **<0.001** | 1.004 | 1.002-1.006 | **<0.001** |
| **Anastomotic time,** minutes^b^ | 1.032 | 0.999-1.067 | 0.057 |  |  |  |
| **Transplant type**, PAK/PTA | 0.534 | 0.232-1.226 | 0.139 |  |  |  |
| **Previous PT** | 0.312 | 0.040-2.431 | 0.266 |  |  |  |
| **Intraoperative transfusions** | 1.182 | 0.655-2.135 | 0.579 |  |  |  |
| **Duration of surgery**, minutes | 1.004 | 1.001-1.008 | **0.019** | – | – | – |
| *BMI*, body mass index; *CI*, confidence interval; *OR*, odds ratio; *PT*, pancreas transplant; *PAK*, pancreas after kidney; *PTA*, pancreas transplant alone. Bold values indicate statistical significance (P<0.05)  ^a^Multivariable analysis adjusted for center  ^b^Available for *n*=247 patients | | | | | | |
